# Methodology for the Development of Augmented Reality Applications: MeDARA. Drone Flight Case Study

**DOI:** 10.3390/s22155664

**Published:** 2022-07-28

**Authors:** Marco Antonio Zamora-Antuñano, Luis F. Luque-Vega, Miriam A. Carlos-Mancilla, Ricardo Hernández-Quesada, Neín Farrera-Vázquez, Rocío Carrasco-Navarro, Carlos Alberto González-Gutiérrez, Yehoshua Aguilar-Molina

**Affiliations:** 1 Centro de Investigación, Innovación y Desarrollo Tecnológico (CIIDETEC-UVM), Universidad del Valle de México, Querétaro 76230, Querétaro, Mexico; marco.zamora@uvmnet.edu (M.A.Z.-A.); calberto.gonzalez@uvmnet.edu (C.A.G.-G.); 2 Centro de Investigación, Innovación y Desarrollo Tecnológico (CIIDETEC-UVM), Universidad del Valle de México, Tlaquepaque 45601, Jalisco, Mexico; luis.luque@uvmnet.edu (L.F.L.-V.); miriam_carlos@my.uvm.edu.mx (M.A.C.-M.); 3 Engineering Area, Universidad Autónoma del Estado de Mexico, Vista Hermosa, Zumpango de Ocampo 55600, Estado de México, Mexico; ohernandezq001@alumno.uaemex.mx; 4 Centro de Investigación, Innovación y Desarrollo Tecnológico (CIIDETEC-UVM), Universidad del Valle de México, Tuxtla Gutiérrez 29056, Chiapas, Mexico; nfarrera@uvmnet.edu; 5 Research Laboratory on Optimal Design, Devices and Advanced Materials—OPTIMA, Department of Mathematics and Physics, ITESO, Tlaquepaque 45604, Jalisco, Mexico; rociocarrasco@iteso.mx; 6 Computational Sciences and Engineering Area, Centro Universitario de los Valles, Universidad de Guadalajara, Carretera Guadalajara Km 45.5, Ameca 46600, Jalisco, Mexico

**Keywords:** augmented reality applications, educational mechatronics, intelligent and adaptive learner interfaces, drone simulator, educational games, edutainment and gamification

## Abstract

Industry 4.0 involves various areas of engineering such as advanced robotics, Internet of Things, simulation, and augmented reality, which are focused on the development of smart factories. The present work presents the design and application of the methodology for the development of augmented reality applications (MeDARA) using a concrete, pictorial, and abstract approach with the intention of promoting the knowledge, skills, and attitudes of the students within the conceptual framework of educational mechatronics (EMCF). The flight of a drone is presented as a case study, where the concrete level involves the manipulation of the drone in a simulation; the graphic level requires the elaboration of an experiential storyboard that shows the scenes of the student’s interaction with the drone in the concrete level; and finally, the abstract level involves the planning of user stories and acceptance criteria, the computer design of the drone, the mock-ups of the application, the coding in Unity and Android Studio, and its integration to perform unit and acceptance tests. Finally, evidence of the tests is shown to demonstrate the results of the application of the MeDARA.

## 1. Introduction

With the Fourth Industrial Revolution, the augmented reality (AR) approach allowed new solutions and provided systems with new intelligence capabilities. With this, the representation of information is possible without losing the perception of the real world [1].

**Figure 1:**
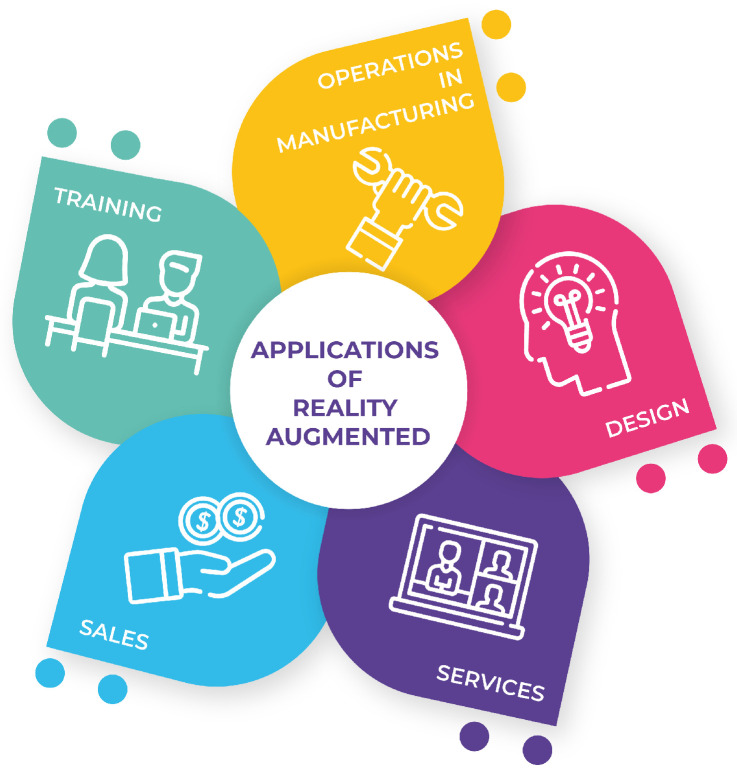
Applications of Augmented Reality in Industry 4.0.

Makhataeva and Varol investigated the main developments in AR technology and the challenges due to camera location issues, environment mapping and registration, AR applications in terms of integration, and subsequent improvements in corresponding fields of robotics [2]. Augmented reality is a technology that complements the perception of and interaction with the world and allows the user to experience a real environment augmented with additional information generated by a computer. It is developed in three phases: (1) In the first, the environment is recognized. (2) Then, the virtual information provided is processed, mixed, and aligned. (3) Finally, the activation is carried out, which is based on the projection of the virtual images. Some of the main applications of augmented reality are in manufacturing operations, design, training, sales, and services (see Figure 1) [3].

In recent years, there has been a rapid growth in the mobile device market that has allowed the emergence of new types of user–machine interaction that are very useful in three-dimensional environments through touch screens. These new possibilities, together with the expansion of computer systems and the appearance of cloud computing, have made possible the appearance of numerous online applications for the design and visualization of three-dimensional models [4].

Liu and Li, in 2021, applied this technology to aerial vehicles to carry out building inspections [5]. Using AR with semiautonomous aerial systems for infrastructure inspection has enabled an extension of human capabilities by improving their ability to access hard-to-reach areas [6]. In Liu and Li, 2021, an AR solution is presented that integrates the UAV inspection workflow with the building information model (BIM) of the building of interest, which is used to direct navigation in conjunction with aerial video during an inspection [5]. Furthermore, remote interactive training of drone flight control with a first-person view is possible through mixed reality systems. A remote trainer carries out the design and management of training scenarios in a virtual environment [7]. The effects of virtual reality, augmented reality, and mixed reality as training enhancement methods are described in Kaplan, 2021 [8].

Augmented reality has been introduced to the educational sector to generate a learning experience that motivates and facilitates interaction with industrial systems that are expensive and complex to acquire since there are institutions with little or no equipment and spaces destined for the new technologies [9,10]. There are several relevant properties and interactions of AR for educational use in learning spaces, in addition to various learning theories such as constructivism, social cognitive theory, makes connections, and activity theory [11].

The main contribution of this work is the design and application of a methodology for developing AR mobile applications that include the concrete, graphic, and abstract levels as part of a macrolearning process. With this, it is possible to generate a new teaching–learning strategy whose key element is the Experiential Storyboard (it is worth mentioning that we are the first to define this new concept) and the test of the developed AR mobile application. Experiential Storyboard establishes that the participant first lives the experience of manipulating a physical and/or virtual object with instructions given in colloquial language. Afterward, a storyboard is generated that includes everything in the user experience and that will be the entrance to the mobile application software development process, which is highly known by the engineering community and combines tools and software for the construction of comprehensive theoretical and practical learning. In addition, this application framework allows the creation of intelligent and safe work environments. This can be implemented in industries and schools that seek continuous personnel training so that they can later manipulate robots and actual machinery without causing damage to the device or damage to the infrastructure or personnel involved.

The paper is organized as follows: Section 2 presents the methodology for the development of augmented reality applications (MeDARA) based on the Educational Mechatronics Conceptual Framework (EMCF), while the application of the MeDARA to the Drone Flight Case Study is in Section 3. Section 4 presents the results of applying the methodology. Section 5 establishes a discussion on the applications of AR in education, and the conclusions close the paper in Section 6.

## 2. Methodology for the Development of Augmented Reality Applications Based on the EMCF

**Figure 2:**
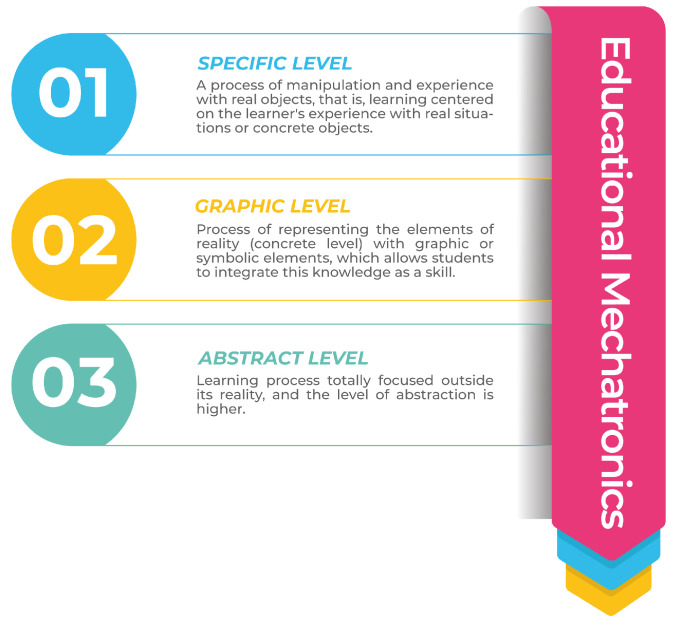
Levels of the learning macroprocess.

This work aims to allow the student to appropriate the ability to use a mechatronic prototype through the MeDARA rather than using an augmented reality app. This methodology focuses on developing augmented reality mobile applications through the three levels of the macrolearning process—concrete, graphic, and abstract—of the EMCF. Its main objective is to develop the knowledge, skills, and attitudes of students to generate solutions with innovative proposals to problems of industrial automation and automatic process control [12] by promoting critical thinking (see Figure 2).

**Figure 3:**
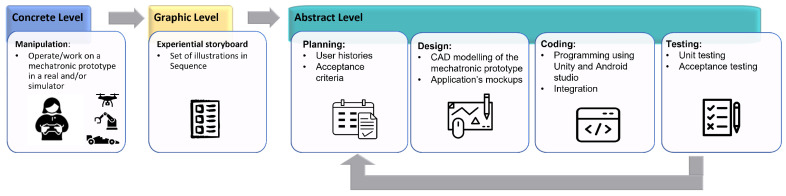
MeDARA for mobile app development.

The concrete level describes the manipulation and experience with real and/or virtual objects for the student to become familiar with the elements of the mechatronic prototype. At the graphic level, elements of reality are presented through graphics or symbols. Finally, the abstract level is a process responsible for obtaining learning outside reality (see Figure 3).

Each of these levels is made up of a set of activities and tasks, which are described below.

### 2.1. Concrete Level

This level aims to allow students to learn to manipulate a mechatronic prototype, either through a simulator or an actual prototype, to become familiar with the concepts and main elements of the prototype—initially, using colloquial language. Some of the examples that can be considered at this level are the manipulation of prototypes that include aerial robots(drones); ground mobile robots (electric vehicles); robot manipulators defined as Selective Compliant Articulated Robot Arm (SCARA); and any physical or virtual device that allows students to visualize elements, movements, and concepts associated with them.

The output of this level is the correct manipulation of the student’s simulator, or the mechatronic prototype, and the recording of each of the associated commands and actions.

### 2.2. Graphic Level

**Figure 4:**
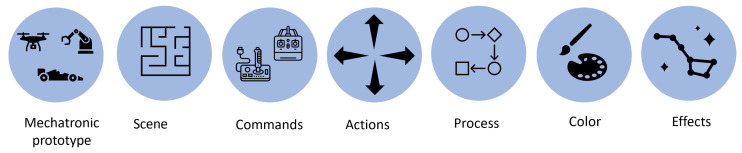
Storyboard elements.

This level’s objective consists of applying the storyboard technique, which has been adapted to create a new concept: the Experiential Storyboard technique. A storyboard is used to show an idea, while an experiential storyboard is used to show an experience. This technique is used to represent the mechatronic prototype and the student’s interactions with it at the concrete level through its representation, based on graphic elements such as the mechatronic prototype, the scenario, commands, actions, process, color, and effects (see Figure 4).

The elements required to carry out this work are described below:

- Mechatronic prototype: This is the object the student will learn to manipulate and/or control. This prototype will allow students to identify its main capabilities and attributes and have a basic overview of its theoretical and practical elements.
- Scenario: Defines the physical environment or place where the student will be interacting with the mechatronic prototype. This place can be outdoors, such as a park, beach, or lake, among others, or an enclosed space, such as a classroom, laboratory, or factory.
- Commands: These are instructions given by the student through a handheld device, called a remote control, which allows maneuvering and making adjustments to the mechatronic prototype. Actions: Describes the linear and/or angular movements performed by the mechatronic prototype based on the commands entered by the student.
- Process: A set of successive phases of a phenomenon or complex fact. Some examples include the phases of flight of a drone, the phases of the trajectory of a car, and the phases of object manipulation by a manipulator’s arm, among others.
- Color: Describes the colors to be assigned to the mechatronic prototype, environment, commands, actions, and the process in general.
- Effects: They add a special sparkle to the storyboard as they describe achievements or highlight the importance of some model elements.

The experimental script describes the history of the student’s relationship with the mechatronic prototype. In this, each command and action associated are shown. Besides, it also presents the object’s before and after, illustrated once the action is performed. Additionally, there is a section of notes, which, if necessary, can describe some aspects of the prototype.

**Figure 5:**
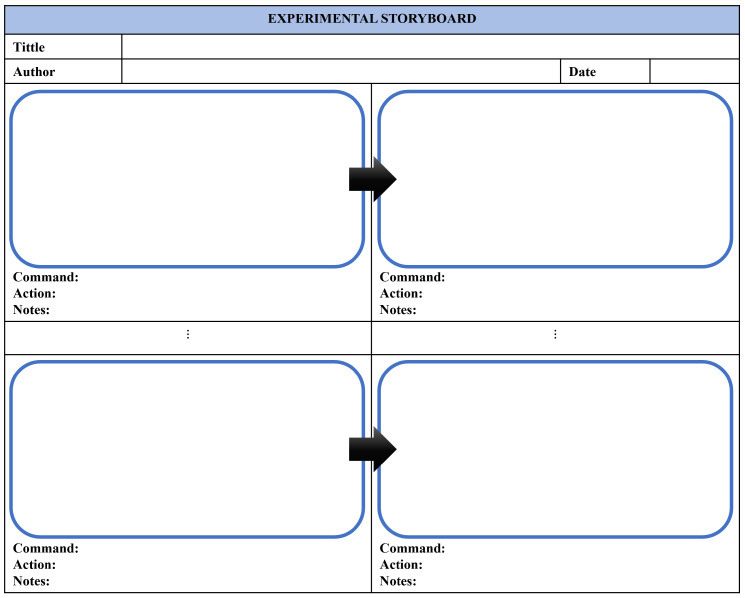
Experiential Storyboard.

For each practice, as many scripts of experiential graphics as necessary will be made (see Figure 5).

It is essential to mention that the graphical elements defined at this stage also form a vital part of the requirements used in the planning at the abstract level. A requirement is defined as required hardware, software, and design elements used before, during, and after the planning and implementation of the practice, software, or project. These requirements are indispensable to ensure the application’s excellent design, visualization, and performance. The output of this level will be the experiential storyboard containing the scenes resulting from each of the commands and actions necessary to carry out the process established for the mechatronic prototype.

**Figure 6:**
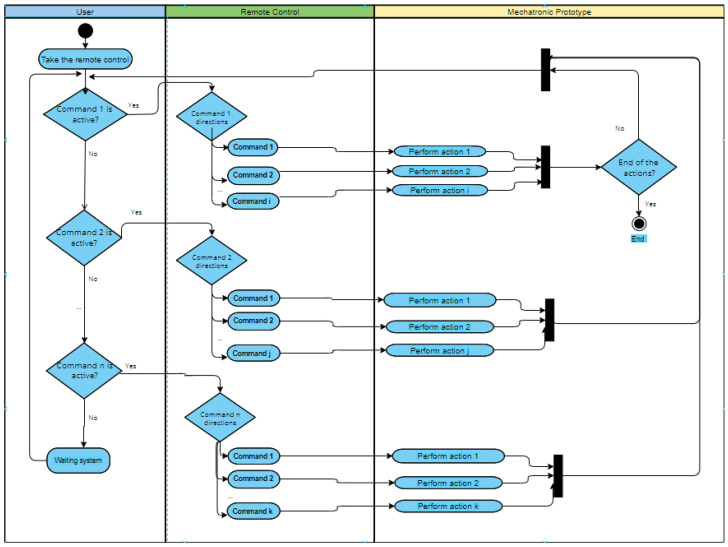
Activity diagram of the actions of the mechatronic prototype depending on the input commands.

Figure 6 depicts a general diagram of activities that describes the sequence of actions or movements executed according to the object used in practice. In the diagram, there is a remote control for every object with which it is possible to enter commands and the set of defined movements or actions, which is essential at this graphic level.

### 2.3. Abstract Level

A study of the various methods or methodologies for developing applications with augmented reality is carried out for the abstract level. This level mainly involves agile methodologies due to the project time and their ease of use; for example, in Syahidi et al., 2021, the development of an application is proposed to facilitate the learning of automotive engineering with the implementation of an application called AUTOC-AR created in augmented reality. The implementation of this application was made using the extreme programming (XP) methodology [13]. XP is an agile software development methodology that aims to produce efficient, low-risk, flexible, predictable, scientific, and distinguishable implementations [14]. XP is also referred to as the new way to develop software. Augmented reality and extreme programming are two techniques that go hand in hand when it comes to educational models, such as applications in tourism [15], health [16,17], and preschool education [18].

**Figure 7:**
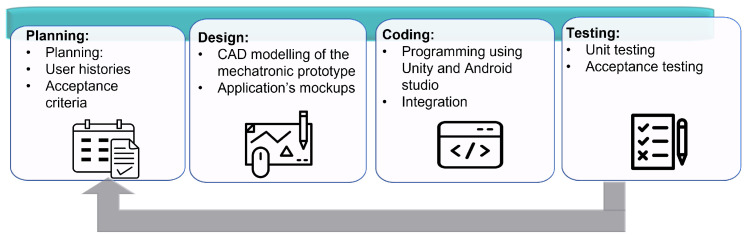
XP Methodology stages.

The methodology chosen for this work consists of four development stages: planning, design, coding, and testing (Extreme Programming, 1996) (see Figure 7). This methodology works using iterations, and at the end of each iteration, functional deliverables are generated that can be used as terminals. It is possible to work from 1 to N iterations. Each of the stages is described below.

Planning. In this stage, a plan must be developed according to the criteria required for developing the software, app, or project. In this stage, the costs and estimated times are also defined through the development of various activities; among them, are the following:

- User stories: They present a description of the system’s behavior and represent the program’s main characteristics and the release plan. User stories are actions that can be performed by the user/student within the software/application or project. These stories are described in conjunction with the associated teacher/advisor in order to make clear the specifications that the application contains.
- Acceptance criteria: This refers to the survey of requirements validated in the testing stage. These criteria describe each requirement that the system or application must meet before the application is released. Some of the requirements that can be considered for the development of the application are the size of the object in augmented reality, colors of the object, button functionality, button position, and filling in requested documents.

Other activities carried out in the planning stage are the delivery plan, number of iterations, and the planning of meetings to visualize the application’s progress. The planning considers the development of an application with augmented reality using the students’ experience with the development of applications based on Unity and Android Studio.

**Figure 8:**
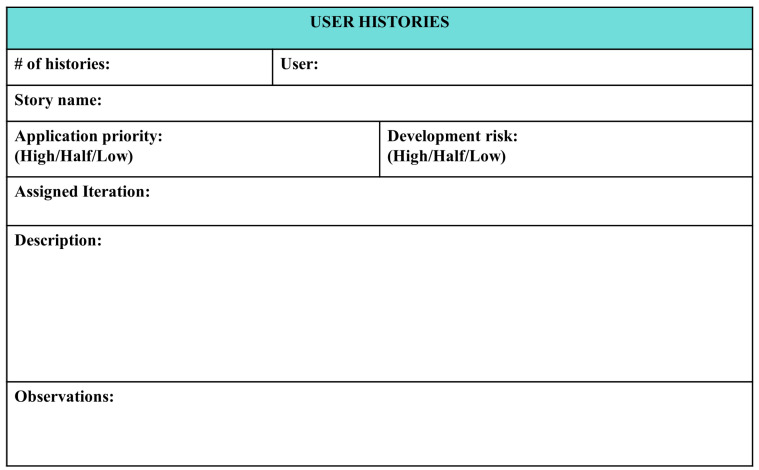
User history.

The user story presented in Figure 8 shows the elements: number of stories; name of the story; user type; the priority in the application; and the risk in the development, classifying them as low, medium, or high. In addition, the iteration number being worked on is specified along with a description of the user story’s process. Finally, observations and their requirements can be completed.

Design. The design work involves the creation of an object-type file of the mechatronic prototype that can be carried out with several CAD software such as SolidWorks, Fusion 360, and CorelCAD, among others. Moreover, for the mock-up creation of a mobile application, the software that can be used are Figma, Balsamiq, Marvel, and Mockplus, among others. Furthermore, any modeling software or tools, such as UML modeling, Class Diagrams, Flow Charts, and others, can be used for the design procedure. It is worthwhile to mention that the main goals are simple concepts and spike solutions, based on the information developed in the planning stage.

Coding. Extreme programming is a methodology that guarantees a user-friendly, easy-to-implement, fast, and dynamic tool. It allows shared work to be carried out with a connection to the client and the developer to improve the implementation of systems [19]. In addition to its easy adaptation, it is also a programming-oriented approach for producers and users of the software, in addition to being one of the recommended methodologies as best practices for software development.

In this stage, a concept known as pair programming is worked on. This refers to communication with all those involved depending on the size of the software, the project to be developed, and the collective ownership of the code, among other aspects. For this project, in the MeDARA, the clients are the students who make the implementation. This allows experimentation with the mechatronic prototype to define the user stories at a specific level, and researchers and teachers accept them.

Tests. This stage helps with the detection and correction of errors in each of the user stories. These tests are carried out before the project’s launch, implying that the programming is terminated once it has been verified that the application works. The tests that are carried out before the release of the project include the following:

- Unit tests: These tests are conducted for each component of the stories to verify and validate their operation according to the requirements specified in the planning. For example, a unit test on the remote control command applied to the mechatronic prototype should produce a specific movement. These tests ensure the correct scaling of the prototype when it is necessary.
- Acceptance tests: These tests are carried out once; the program certainly works. The objective is to validate the acceptance criteria defined in the planning stage by users/students and that everything in the project works correctly. If the project is accepted, it can be considered ready to be used as a training tool for future engineers or moved to the next iteration. If the project is accepted, it is established with the released status. The decision to advance or not to the next iteration can be made depending on whether more elements are able to be added to the application or whether more detail is required for the scene where the user interacts. All modules must undergo tests before integration with more iterations or releases. Tests are carried out at different stages of software development, and these can be documented tests or small tests of code functionality.

## 3. Application of the MeDARA to the Drone Flight Case Study

**Figure 9:**
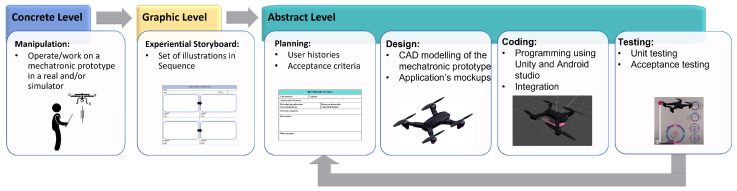
MeDARA applied to a drone flight with Augmented Reality.

For the application of the MeDARA, the subject selected incorporates robotics as the first case of an application using the flight of a drone. A set of activities and necessary tasks were carried out for each of the steps that make up the methodology (Figure 9). As previously described, the methodology is composed of three main levels: concrete, graphic, and abstract.

### 3.1. Concrete Level

**Figure 10:**
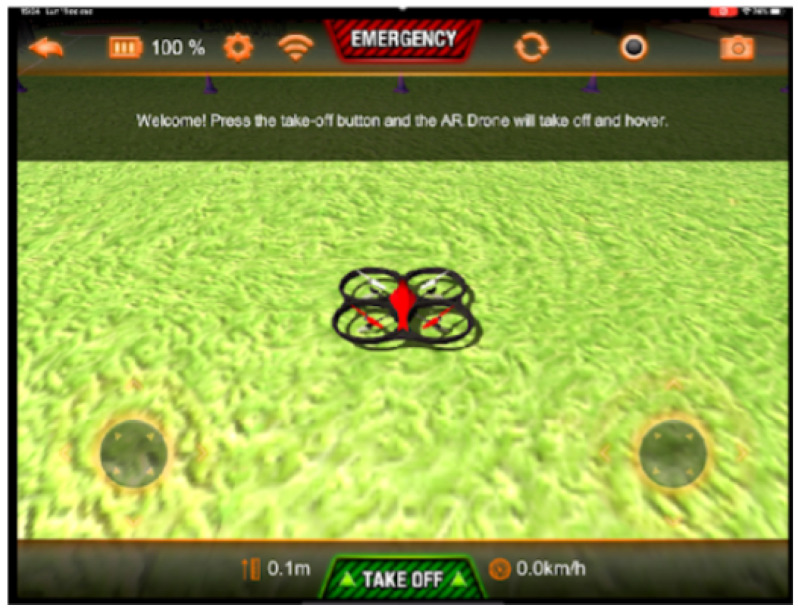
Software AR Drone Sim Pro Lite.

The objective of this level is to manipulate an actual prototype or a simulator according to the type of software that will be developed. For this particular project, it is recommended at this stage to use the AR Drone Sim Pro Lite simulator software (Figure 10), which can be installed on a mobile device. Students identify the drone flight phases: takeoff, flight, and landing.

- **Takeoff:** the drone rises to a certain altitude;
- **Operational flight:** the drone can hold a stationary position in the air (hover) and maneuver flight where mixed movements to the left, right, forward, backward, up, and down are possible;
- **Landing:** the drone landing gear makes contact with the ground.

**Figure 11:**
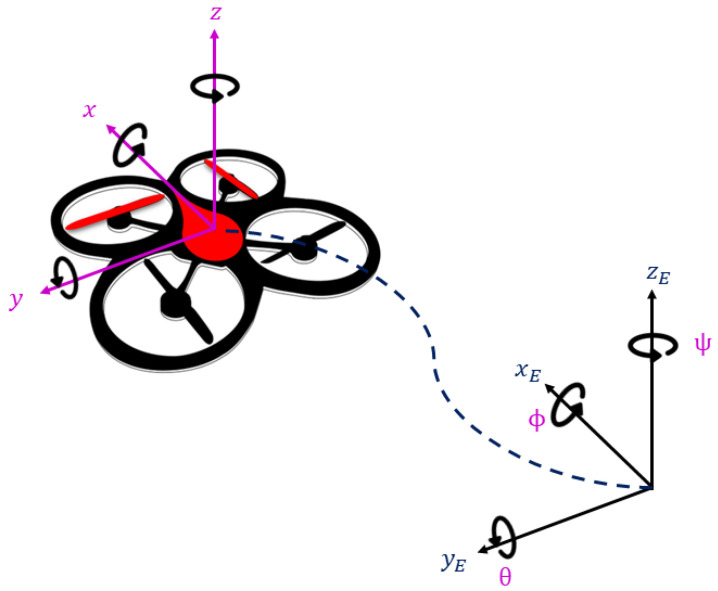
Drone coordinate systems.

The angular and linear position of the drone with reference to the Earth-fixed frame $E^{E}\left(O_{E},x_{E},y_{E},z_{E}\right)$ can be seen in Figure 11. The absolute position of the drone expressed in $E^{E}$ is described by x,y,z position, and its attitude by the Euler angles ϕ,θ,ψ, referring to roll, pitch, and yaw angles, respectively.

It is worthwhile to mention that this work uses colloquial language since the focus is to develop the mobile application, not the drone concepts. For more reference on formal language when dealing with drones and its mathematical model, see [20]. Moreover, a drone flight instructional design can be found in [21].

### 3.2. Graphic Level

**Figure 12:**
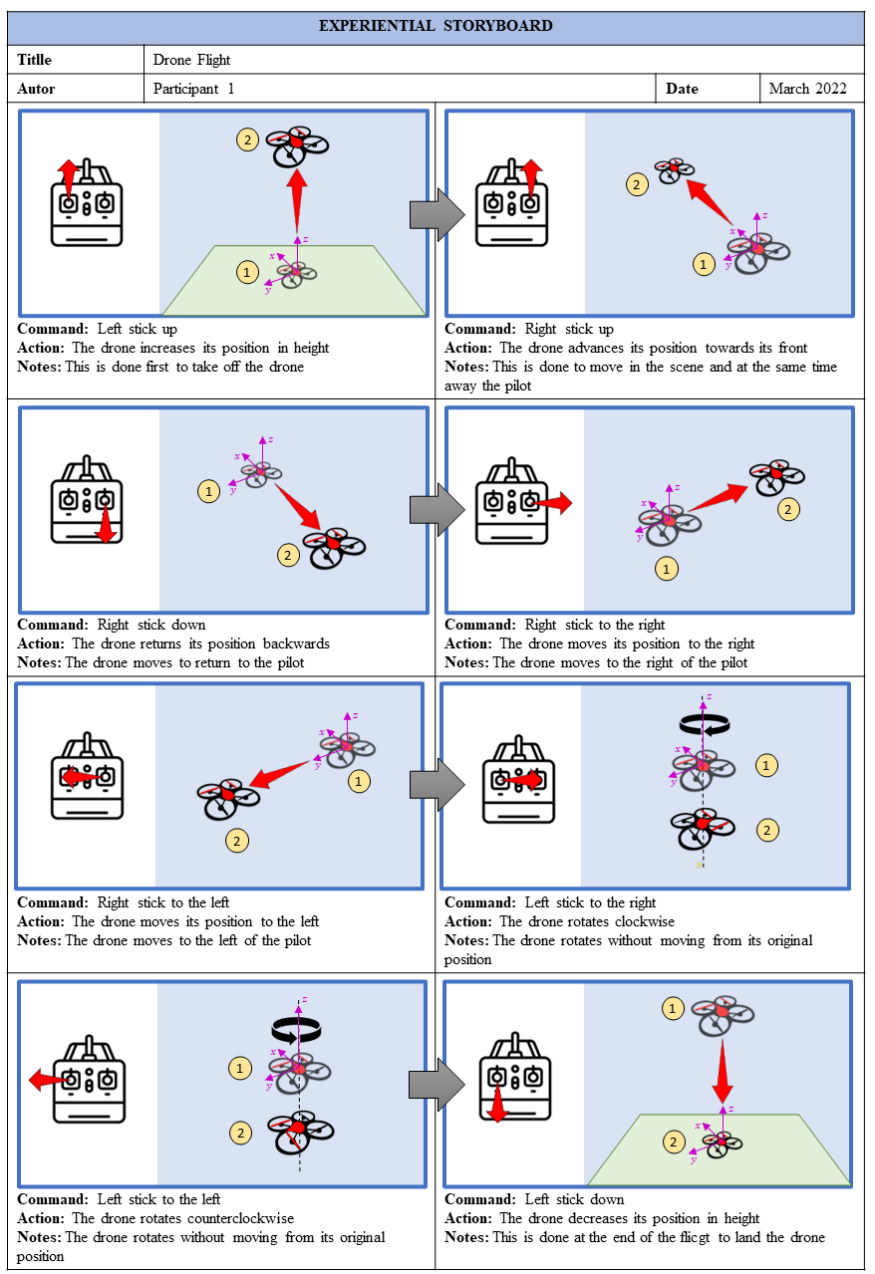
Storyboard containing the commands and actions of the drone flight.

The objective of this level is for users to identify the elements with which they interacted in the first level. For this, the experiential storyboard technique is applied. The results are shown in Figure 12, which describes in detail how each of the commands is carried out in the simulator, and the produced effects (actions) can be seen. The initial and final positions are represented graphically after the execution of the specific control command. For example, if the command is left stick up, the action is for the drone to increase its position in height (altitude position). Moreover, a note mentions that the drone lifts off the ground (takeoff). Then, the subsequent commands can be executed from the previous command. Students must fill out this storyboard to identify the movements made before, during, and at the end of the drone’s flight.

**Figure 13:**
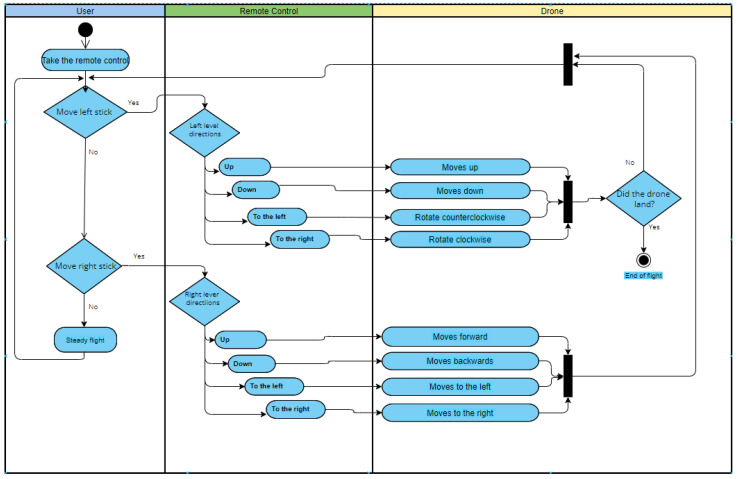
Activity diagram of drone actions based on remote control input commands.

As an example, Figure 13 is presented, where the activity diagram for the drone flight activity is shown. It can be noted that the user manipulates the remote control through the left and right sticks. Each stick has the associated commands: up, down, to the left, and to the right. Each of these commands has an associated set of actions: for example, if the right stick is moved up, the drone rises (increase its altitude position); on the other hand, if the right stick is moved to the right, the drone moves to the right (longitudinal position), until the user stops performing the action.

According to abstract programming, the object is defined as an abstract entity. The set of movements is the abstraction of actions. Each of the movements made by the drone is an abstraction of the virtual machine, defined as the extent to which the drone increases and/or decreases its altitude each time these buttons are pressed.

Once this storyboard and the activity diagram are completed, the next step is the abstract level.

### 3.3. Abstract Level

This level considers the development of an application for the simulation of drone flight in augmented reality, which applies each of the learning obtained in the two previous levels. A set of software and hardware requirements were necessary to develop this AR prototype. More details are described and presented in Table 1.

#### 3.3.1. Planning

User story tasks and the realization of the acceptance criteria are included in the activities of the students.

**Figure 14:**
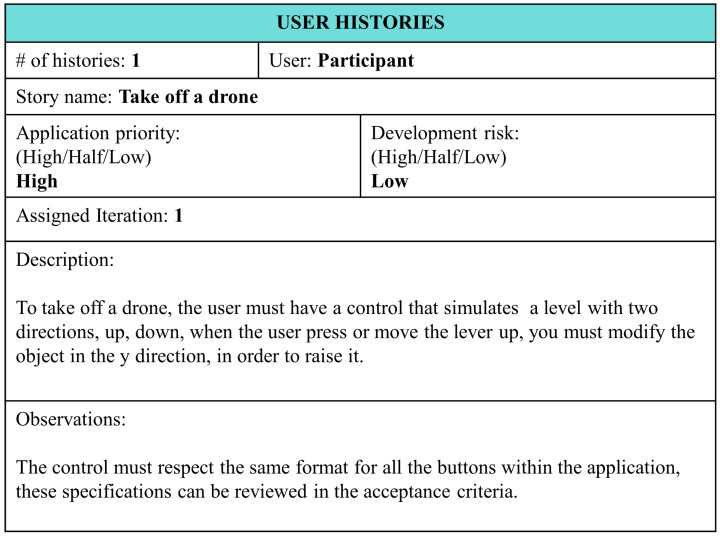
User stories for a drone takeoff.

- User stories
  This involves filling out a template that indicates every action that can be performed by users in the AR application, such as takeoff and landing of the drone; moving it up, down, left, and right; and rotating clockwise and counter-clockwise. Every action presented in the Experiential Storyboard has a related user history (see Figure 14).
- Criteria of acceptance
  These need to be specified at the beginning of the creation of the software and include the following:
  – Drone size in augmented reality (*scale X = 4.13, Y = 4.13,Z = 4.13*);
  – Drone design colors (*black, pink, blue*);
  – Number of movements allowed for the drone (*left, right, up, down*);
  – Verification of the commands up, down, left, right, turns, etc.;
  – Design, position, and size of the buttons.

#### 3.3.2. Design

**Figure 15:**
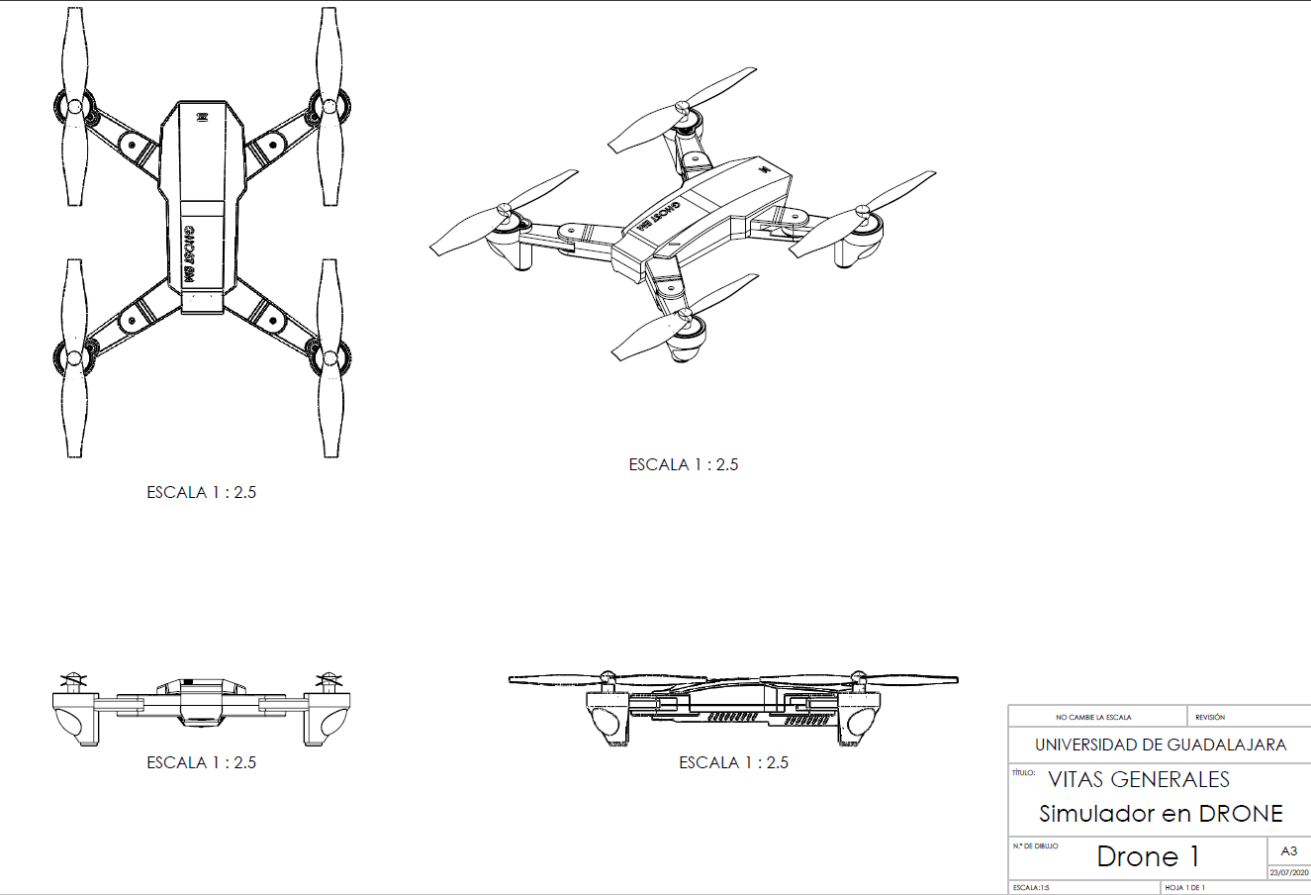
Isometric view with details.

Drone design is performed with computer-aided design (CAD) software: considering the conceptual design of the drone oriented towards educational mechatronics, the design and assembly of the parts were carried out to form the drone model in the Solidworks 3D modeling software. Figure 15 illustrates the isometric view of the drone with the materials’ details; the assignment of the color palette; and the isometric, top, side, and front views with the general measurements of the object.

**Figure 16:**
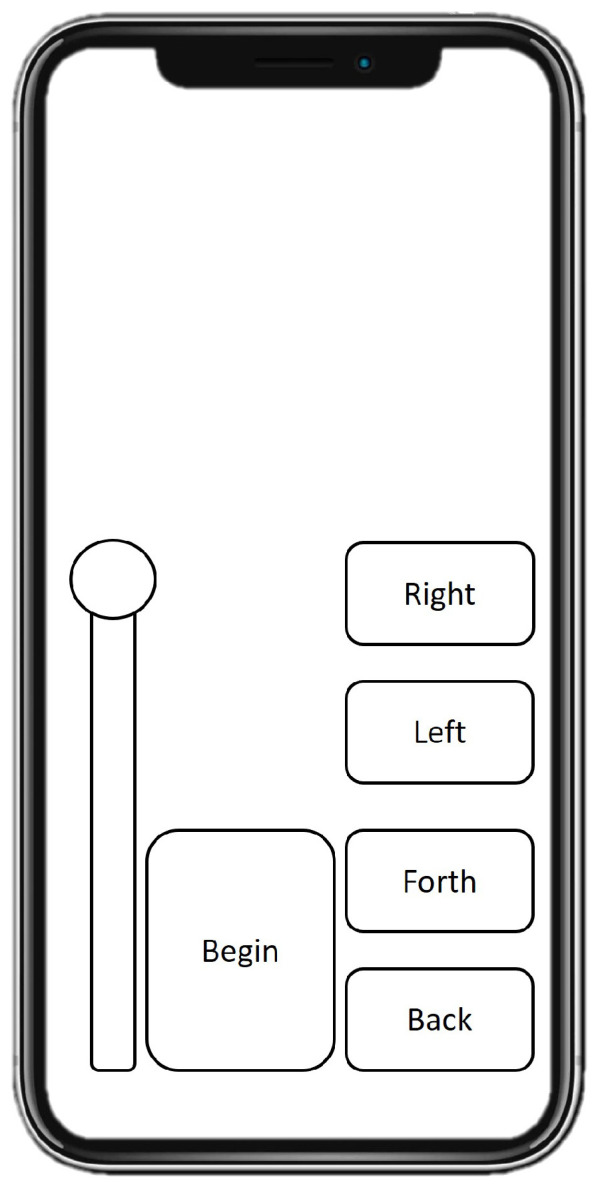
Mock-up for the augmented reality application.

Then, a mock-up of the mobile application is performed to design the scale and position of every button (see Figure 16). A mock-up represents the prototype of the project to be carried out. The slider gain increases or decreases the drone’s altitude, while the right, left, forth, and back buttons command the right, left, forth, and back movements, respectively.

#### 3.3.3. Coding

**Figure 17:**
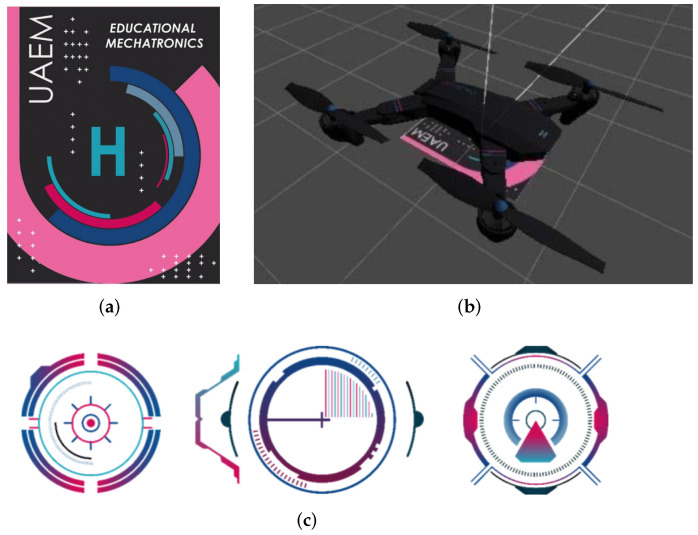
MOCAP measurements of real flight with drone following the second movement. (**a**) Target to activate the augmented reality model. (**b**) Drone in the Unity engine. (**c**) Command buttons in the AR application.

Once the 3D model of the drone was obtained, it was exported to the Unity engine, which allows the development of applications for augmented reality. Simultaneously, the Vuforia engine runs and acts as a compliment that generates the graphics in augmented reality in a mobile device (Figure 17). The image that simulates a heliport to be recognized by the mobile device can be seen in Figure 17a. Then, the activation of the drone model in augmented reality is sent to show the drone virtually (see Figure 17b). Finally, the on–off and command buttons of the drone are shown in Figure 17c.

Subsequently, the Unity mobile application generated the codes of the different buttons that make up the drone’s movements in the AR.

**Control Pseudocode**

To develop the pseudocode (Algorithm 1) used to control the drone, the variables associated with the starting speed, rotation, elevation, or height and the movement of the four drone propellers are initialized. Subsequently, Boolean values related to the movements allowed in the drone are declared, such as moving forward (moveForward), movement backward (moveBack), movement to the right (moveRight), movement to the left (moveLeft), control and movement, and starts the check. Then, the movement variables are set to false in order to be able to perform the actions later, which are described in Algorithm 2.
**Algorithm 1** Initialization of variables for the drone movement.**Input:***Velocity*, *VelocityH*, *VelocityRot*, *Propeller1*, *Propeller2*, *Propeller3*, *Propeller4***Output:***Boolean with movement*  1: Initialization of input variables  2: Boolean declaration variables: Adelante, moverAtras, moverDerecha, moverIzquierda, Start, StartControl  3: Start  4: Initialization of Boolean variables to false

Algorithm 1. Initialization of variables for the drone movement. Input: Velocity, Velocityh, VelocityRot, Helix1, Helix2, Helix3, Helix4. Output: Boolean with movement.

1. First, the input variables are declared. 2. Then, a declaration of Boolean variables such as: moveForward, moveBack, moveRight, moveLeft, start, startControl are required. 3.Finally, when all parameters are ready, the prototype starts. 4. Then, the initialization of Boolean variables in false is defined before to start any movement.

As it is mentioned before Algorithm 2 describes the movements forward, up, left, and right. For this purpose, it is verified if the control is pressed; if so, the Boolean variable associated with the action changes to true until the control is released; then, the variable changes to false and stops acting. Each movement has the same behavior associated with it.

With this, the first augmented reality mobile application integration is completed and the MeDARA continues with the testing stage.

For more details, please visit the following page: https://bit.ly/3jRvmIu (accessed on 15 June 2022).
**Algorithm 2** Drone actions**Input:***Velocity*, *VelocityH*, *VelocityRot*, *Propeller1*, *Propeller2*, *Propeller3*, *Propeller4***Output:***Boolean with movement*       **if** Adelante isPressed=true **then**  2:        moverAdelante=true       **end if**  4:  **if** Adelante isRelease=true **then**             moverAdelante=false  6:  **end if**       **if** moverAtras isPressed=true **then**  8:        moverAtras=true       **end if** 10: **if** moverAtras isRelease=true **then**             moverAtras=false 12: **end if**       **if** moverIzquierda isPressed=true **then** 14:       moverIzquierda=true       **end if** 16: **if** moverIzquierda isRelease=true **then**             moverIzquierda=false 18: **end if**       **if** moverDerecha isPressed=true **then** 20:       moverDerecha=true       **end if** 22: **if** moverDerecha isRelease=true **then**             moverDerecha=false 24: **end if**

#### 3.3.4. Testing

The testing stage is performed to obtain a final functional model. Some metrics are defined to model and evaluate the complete AR prototype. The basic AR metrics can be found in Table 2. These metrics ensure the proper functionality of the applications throughout the whole implementation. Some of them are mentioned as follows.

The time spent on the app was tested at different moments in the development process. It was intended to define the experience of the application between 5 and 15 min per user, where all movements allowed in the drone were put into practice that understood the context of each user’s augmented reality. This time is equivalent to a commercial drone’s time autonomy. In addition, the response time of the buttons in the application is defined as 0.05 s, in order to provide a realistic experience of the movements of a real drone.

Regarding the quality of the images used for the project development, these were exported in PNG format to show transparency at the launch of the application and give it that futuristic look that goes with the static of the drone. Further, the images maintain an approximate weight of 156 KB, with dimensions of 2829×1830 pixels, using a depth of 32 bit to display a defined and easy-to-load image. As for the three-dimensional model that is the drone, it is kept in .fbx format, which is a 3D object.

The application was tested several times at different moments of the day. On all occasions, the application was available without failures during the final testing. The application is currently located locally, and all those users who want to use it must request permission from the administrator until it is released in the Android store.

The application went through different stages of testing throughout its development. Some of the remarkable configurations that were improved include the following:

* It was proven that the propellers work correctly and that they do not remain suspended in the air when turning.
* Tests were carried out to configure the revolutions of the drone’s movement so that they would not be seen as static and their movements would imitate real drones.
* The application was tested to ensure its availability at any time. It is worth mentioning that all functionalities work correctly.

For the drone model with augmented reality, the tests were divided into unit and acceptance tests; the following is a description of each of them.

**Figure 18:**
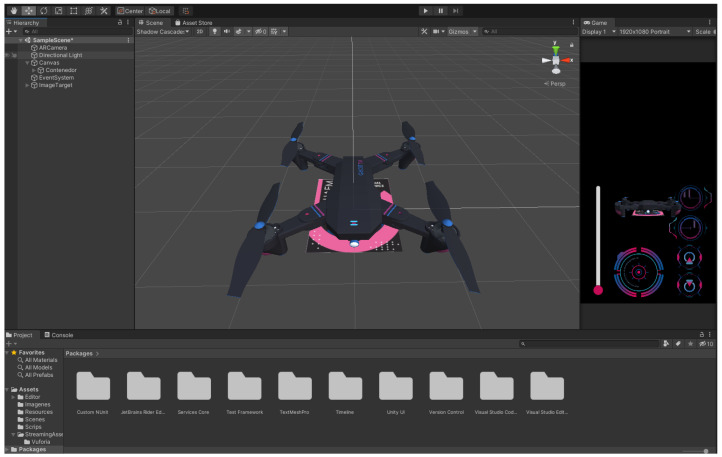
Design of the drone environment in augmented reality using Unity.

**Figure 19:**
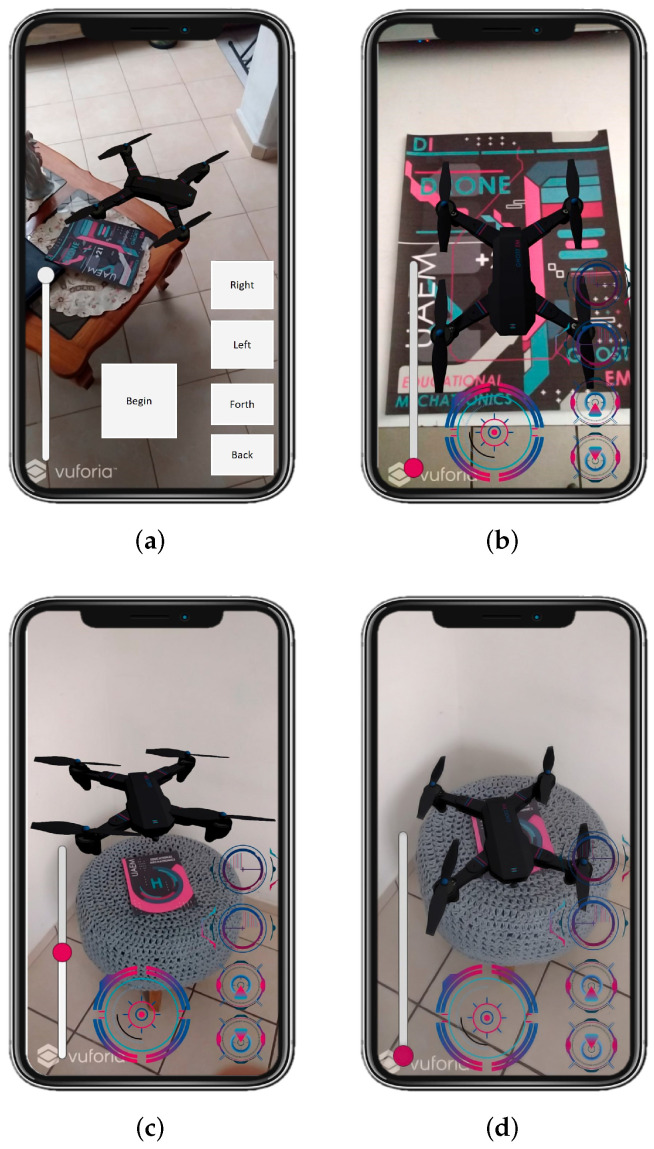
Different views of the application tests. (**a**) Design of the drone environment in augmented reality using Unity. (**b**) Display of image on device’s buttons in the design for Android devices and integration of the buttons. (**c**) Unit tests of each of the buttons and their operation. (**d**) Prototype functionality test.

**Figure 20:**
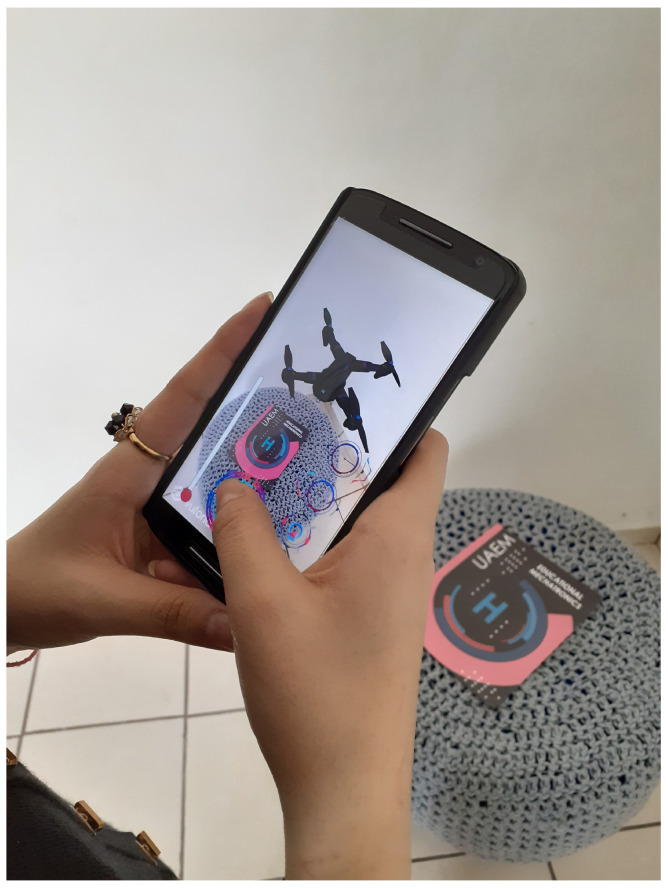
Prototype functionality tests and mobile application.

- Unit tests
  These contribute to verifying and validating each aspect of the augmented reality model, the buttons’ functionality, the drone’s size, and the Unity design of the drone. The tests are presented in more detail below. Unit tests of the static Augmented Reality (AR) prototype are of the Unity simulator.
  Figure 18 presents the complete design with the drone integrated into the platform for the specific tests. We validated the drone’s size within the application, the design colors, and the platform’s scale where it lands. We also validated the user’s view when the model was displayed on a mobile device.
  In this unit test, several cases were applied to visualize how the application and functionality were being integrated into the complete AR model. Some of these tests include the following:
  – Tests of the deployed static model
    This test validates the illustration displayed when the model is activated (Figure 19a). It was performed with the Vuforia add-on using a mobile device preview. At this stage, the design of the buttons had not been added yet, only the position of the buttons was validated within the image display on the mobile device. Designers carry out this test in the development of the application.
  – Testing of the AR kinematic model
    This test validates the AR kinematic prototype using a mobile device preview with the Vuforia application. The application deployment was performed for Android-based mobile devices. The colorimetry for the buttons and the space allocated for the buttons was also validated, see Figure 19b, while Figure 19c displays the close-up model already with the buttons integrated and the final colors.
  – Testing of the dynamic prototype using AR input commands
    In these tests, the final prototype is shown to be working. This test aims to validate each of the application’s buttons, including the final design. For this, each of the buttons were tested to see the type of movement, controls, and actions on the drone. Its operation was validated and accepted. Figure 19d shows one of the tests performed.
- Acceptance test
  Once the unit tests were done, the design and the results of the programming environment were validated. Acceptance tests help to determine if changes are made within the design. This stage includes the approval of the people involved so that the application can be defined as completed. This kind of test is made by users who give feedback on the app’s functionality. The acceptance tests that were performed are described below.
  – Acceptance test for the deployment of the drone using a mobile device This test validates and verifies the app’s compatibility on a real mobile device. The interface and interaction with the drone were finalized intuitively and successfully, keeping the cyberpunk design and unifying all the application components.
  – Testing of controls using the mobile application The final tests considered the interaction of a user with the application. For this purpose, the complete application runs on a mobile device. Figure 20 shows the image of a user interacting with the dynamic prototype using AR input commands on the mobile device.
  – Additional test
    (1) A first test was performed where the drone must appear when the target is scanned.
    (2) The buttons that perform the movements to the right, left, back, and forth were tested as well (see https://n9.cl/fdt8v (accessed on June 15 2022)).
    (3) In addition, another test showed when the mobile device was moved away from the target (see https://n9.cl/werqp (accessed on June 15 2022)).
    (4) Demonstrated that the drone still works even though the mobile device stops seeing the target (see https://n9.cl/v9z6y (accessed on June 15 2022)).

## 4. Results

In order to establish a mathematical framework, the descriptive statistical analysis of the initial contact assessment for the augmented reality mobile application test for the experimental and control group is performed. It is worth mentioning that data consider the participant’s necessary time to understand the operation of the mobile application. The *t*-test is used to determine if there is a significant difference between the means of the two groups. The hypotheses tested by the independent samples *t*-test are

$$\begin{array}{c} H_{0}:\mathrm{there}\;\mathrm{are}\;\mathrm{no}\;\mathrm{differences}\;\mathrm{between}\;\mathrm{the}\;\mathrm{means}:\mu_{x}=\mu_{y}\\ H_{1}:\mathrm{if}\;\mathrm{there}\;\mathrm{are}\;\mathrm{differences}\;\mathrm{between}\;\mathrm{the}\;\mathrm{means}:\mu_{x}\neq \mu_{y} \end{array}$$

The conditions of an independent samples *t*-test for hypothesis testing are Independence, Normality, and Homoscedasticity.

The Shapiro–Wilk test finds significant evidence that the data come from populations with a normal distribution (see Table 3).

Several tests allow comparing variances. Since the normality criterion is met, one of the recommended tests is the Bartlett test. Table 4 shows that no significant evidence is found (for alpha = 0.05) and that the variances are equal between both populations.

Therefore, the *t*-test with Welch’s correction must be performed. The results are shown in Table 5 where Dof is the abbreviation of Degrees of freedom.

Given that the *p*-value ($1.28132\times 10^{-6}$) is less than the alpha level of significance (0.05), there is sufficient evidence to consider that there is a fundamental difference between the learning time of the application keypad of the individuals of the control group and individuals in the experimental group. The effect size measured by Cohen’s d is large (1.9577).

## 5. Discussion

The object of analysis presents the principles of Industry 4.0 expressively in higher education courses described in the description of the professional profile, field of activity, or curriculum and the assumptions are intertwined as a pedagogical proposal [22].

As Atamanczuc and Siatkowski (2019) point out, changes in the world of work have led to greater precariousness in working conditions and labor relations, as well as in the lives of workers. However, this is not announced in the principles of the so-called Industrial Revolution [23]. It is necessary, therefore, to reflect on the impacts of this new “industrial revolution” on the increase in productive capacity and the possibilities of emancipation or the subordination of workers.

It is possible to understand the learning itinerary as a route in which the user can learn specific material. Its approach has been expressed in terms of a guided visit of learning material [24]; a formative structure providing open and dynamic processes [25]; a guide on how students learn the content [26]; and the knowledge organizers of teachers and students and sequencing of content that fits the student’s profile [27].

Considering the impact on the students’ education, certain personal learning itineraries have been explained in various studies, which highlight different conclusive aspects such as the following: allowing the teacher and the students to have real control in the subject organization [24]; implementing learning itineraries to improve student perception of the classes [24]; using learning itineraries in a linear or flexible way, favoring the teaching–learning process [12,18]. Note that the flexible learning design requires teaching competencies and induction processes regarding the technological mediation used for students [26,28].

The implementation of robotics and computational thinking in education and the decision to include robotics and PC content is not neutral but, rather, has evident political–economic motivations, such as the following:

- Encouragement of more technical, computerized, and specialized careers (STEM careers);
- Inclusion of business in the educational system through “philanthropy”;
- Increasing incorporation of robots into society;
- Movement of capital from the public to the private sector;
- Normalization, by the education sector, of the company discourse that this “has to be so”;
- Involvement of companies, through concrete projects, in academic life.

The forms of knowledge representation used by the students to solve problems according to their cognitive style are not exclusive. They only evidence the preference for the forms of codification that, according to their dimension, generate information recall. From this perspective, it is important to emphasize that the context of the subject is technical; therefore, it favors the use of representations based on artifacts. From the point of view of navigation in the pathway, because it configures inputs, it delivers complete control to the learner, and the teacher configures their role as the mediator between the pathway and the learner [29]. Regarding the learning outcome, the study has revealed a relationship between the implementation of the learning itinerary, mediated by AR, for the mechatronics course and the learning outcomes [30,31]. Finally, it is essential to emphasize the contribution of this research in terms of scientific references that establish a relationship between the use of personal learning itineraries and augmented reality in the training of students, where academic performance is improved in addition to the research process. For future work, incorporating mixed reality and extending the applications are proposed.

## 6. Conclusions

Implementing the MeDARA through the three levels of macrolearning of the EMCF—concrete, graphic, and abstract—shows its effectiveness. The student was capable of developing an AR mobile application using an existing drone flight simulator app, the experiential storyboard, and the programming tools.

The final functional model was verified when implementing the tests, within which unit and acceptance tests were performed. Each of the model’s aspects in augmented reality, the buttons’ functionality, the drone’s size, and design in Unity of the drone were validated, while the acceptance tests determined if changes were made within the design.

In addition, the results show that when the initial contact assessment with the developed AR mobile application occurs, there is a real difference between the learning time of the application buttons of the control group and individuals in the experimental group. This difference means that the buttons in the AR mobile application can be improved to make it more intuitive to the users.

The present innovation used in augmented reality for education corresponds to the process type since the proposal offers a form of teaching that differs from other educational proposals. Incorporating augmented reality in the learning process is innovative because it implies a paradigm shift in how learning is approached through the implementation of the EMCF—incorporating technologies as tools that support the process of academic formation. Moreover, AR mobile applications can be used to simulate an automation process in the industry.

## Figures and Tables

**Table 1:** Software and hardware requirements.

Software		Hardware	
**PC**	**Mobile**	**PC**	**Mobile**
Unity Hub 3.0.1	Android version 6.0.1	Core i5 9th generation	RAM free 2 GB
Visual Studio 2015 o posterior		RAM 4 GB+	ROM 16 GB
Android Studio (SDK), Java (JDK)		NVIDIA 512 MB (GTX 650 minimum)	Resolution 1920×1080
Windows 7 SP1+		Disk space 10 GB	Smartphone
AutoCad 2019+			

**Table 2:** Evaluation metrics.

Metrics	Value
Total implementation hours	166
Total methodology implementation hours	226
Overall stage size	250 cm in *X*, *Y*, and *Z* axis
Time spent in the app	5–15 min per user
Image size	156 Kbs
Image pixels	2829×1830 pixels using 32 bits of depth
Image quality	Ultra-quality and full-response texture quality, 2× multisampling antialiasing parameter
Frame rate	60 Frames per second (FPS)
Button response	0.05 s
Availability	The application is available for 24 h but, in a local manner, the app needs to be released to the Android store.

**Table 3:** Shapiro–Wilk normality test.

Initial Evaluation Test	W	*p*-Val	Normal
Experimental	0.963655	0.619153	True
Control	0.968042	0.713114	True

**Table 4:** Bartlett Homoscedasticity test.

	T	*p*-Val	Equal_var
Bartlett	9.308328	0.002281	False

**Table 5:** Welch’s correction test.

	T	Dof	Alternative	*p*-Val	CI95%	Cohen-d	BF10	Power
*t*-test	−6.1909	27.2369	two-sided	1.$23419\times 10^{-6}$	[−19.37,−9.73]	1.95773	$3.603\times 10^{4}$	0.999976

## Data Availability

Not applicable.

## Abbreviations

The following abbreviations are used in this manuscript:

MeDARA	Development of Augmented Reality Applications
EMCF	Educational Mechatronics Conceptual Framework
AR	Augmented Reality
UAV	Unmanned Aerial Vehicle
BIM	Building Information Model
SCARA	Selective Compliant Articulated Robot Arm
AUTOC-AR	Extreme Programming
XP	Extreme Programming
CAD	Computer-Aided Design
UML	Unified Modeling Language
PC	Personal Computer
SDK	Software Development Kit
JDK	Java Development Kit
RAM	Random-Access Memory
MB	Megabyte
GB	Gigabyte
ROM	Read-Only Memory
PNG	Portable Network Graphics
KB	Kilobyte
STEM	Science, Technology, Engineering, and Mathematics
FPS	Frames per second
